# Unilateral Abducens Palsy and Headache in Postpartum Patient Presenting to Ophthalmology

**DOI:** 10.7759/cureus.19968

**Published:** 2021-11-28

**Authors:** Matthew Sikorski, Andreea Ionean

**Affiliations:** 1 Ophthalmology, NHS Grampian, Aberdeen, GBR

**Keywords:** postpartum headache, ophthalmology, orthostatic headaches, abducens palsy, post dural puncture headache

## Abstract

Postpartum patients rarely present to eye casualty. Here we report a case of a seven-day postpartum patient with sudden onset horizontal diplopia and an occipital headache from the perspective of the ophthalmology eye casualty in a tertiary hospital. Intracranial imaging ruled out any acute pathology. The patient required epidural anaesthesia during labour, and a diagnosis of a post-dural puncture headache (PDPH) with an abducens nerve palsy was reached. A blood patch was not provided in this case. The headache settled and the diplopia self-resolved three weeks postpartum. PDPH with extra-ocular muscle paresis is rare, and, as the diplopia onset usually follows the characteristic orthostatic headaches of PDPH, it is likely that these patients are followed up by obstetricians or anaesthetists. These patients rarely present to ophthalmology services to receive this diagnosis, therefore ophthalmologists might not be familiar with this pathology. To our knowledge, this is the first case report of PDPH with cranial nerve palsy that has been documented to present to an ophthalmology department.

## Introduction

Abducens nerve palsy is the most common type of ocular nerve palsy [1], and ophthalmologists are familiar with diagnosing and managing this condition. The incidence of abducens nerve palsy increases with age [1,2], therefore young patients presenting to the ophthalmology services with this condition are rare. While a vascular aetiology is most common in the elderly, in a young population group, other causes are more common [1], and extensive investigations are usually organised to exclude them.

Isolated abducens nerve palsies do not tend to be painful, therefore the presence of associated headaches should be considered as an important sign for intracranial pathology that warrants further investigations. In the immediate postpartum period, headaches are fairly common, majority attributed to primary headaches the most common being migraines [3]. Despite this, a postpartum patient should raise a degree of suspicion. Particularly if the presenting headaches are associated with other neurological “red flag” features, since they could encompass serious pathologies, such as pre-eclampsia, cerebral venous thrombosis, or subdural haematoma [4].

Here we report the case of a postpartum patient who presented to eye casualty with a headache and diplopia caused by intracranial hypotension. We discuss the approach to diagnosis and management options for this condition which, although not uncommon in an anaesthetic service, is rarely associated with diplopia and almost never first presents to ophthalmology.

## Case presentation

A 27-year-old female seven days postpartum presented to her local optician prior to attending eye casualty with a sudden onset horizontal diplopia. The diplopia disappeared upon closing either of her eyes, and it was worse on her left gaze. No other visual disturbances were noted apart from the sudden onset of horizontal diplopia, which was constant.

The patient was fit and well and had no past medical history. Her pregnancy was eventless apart from prolonged labour requiring an epidural anaesthetic, followed by uneventful forceps delivery. Shortly after delivery, the patient developed occipital headaches and neck pain but was subsequently discharged from the hospital with caffeine tablets. These tablets settled the pain initially, but over a period of days, the headaches became more frequent and intense. The headaches worsened when upright and alleviated, when prone. She denied any nausea, vomiting, loss of consciousness, seizures, photophobia or pyrexia. The headaches settled on the morning of her attending the eye clinic (seventh day post-partum).

Visual acuity was 6/4.8 in both eyes. Intraocular pressure was 9 mmHg in the right eye and 10 mmHg in the left eye. No relative afferent pupillary defect (RAPD) was detected and her colour vision was full in both eyes using Ishihara plates. Humphrey central 24-2 threshold visual field was full and the blind spot was not enlarged. Slit-lamp examination was unremarkable in both eyes. Examination of the cranial nerves elicited a left abducens nerve palsy, with the left eye not being able to abduct past the midline. No proptosis was noted. No other focal neurology was present.

The patient was clinically stable. Her blood pressure was 107/73 mmHg with a heart rate within the normal range. Full blood count, urea and electrolytes, liver function tests, and C-reactive protein were normal. The urine dipstick showed no protein. CT head revealed a slightly prominent pituitary gland at 9 mm diameter, but no acute intracranial pathology or intracranial masses were detected. 

In view of her labour history, post-dural puncture headache (PDPH) with extraocular muscle paresis was suspected and the case was discussed with the duty epiduralist who admitted the patient following assessment at maternity triage.

An epidural blood patch was not offered by the anaesthetists in this case, as it was considered for it to be unlikely of any benefit as the neuropraxia would have already occurred and the dural puncture would likely have healed. To exclude a persistent dural leak (wherein a blood patch might help in reducing further damage to the abducens nerve), an MRI head and spine with gadolinium contrast was suggested by the radiologist, but the patient declined the scan. 

The patient was treated conservatively with bed rest, hydration and caffeine tablets, and eventually discharged from the hospital with follow-up by anaesthetics and ophthalmology. From an ophthalmology point of view, an eye patch was offered to alleviate the patient's diplopia. Her visual symptoms and headache gradually settled over two weeks and the abducens nerve palsy completely resolved when attending her follow-up within the orthoptic clinic. The patient was discharged from further follow-up.

## Discussion

Headaches are common in the postpartum period and are often attributed to primary headaches, particularly migraine and tension-like headaches, which make up 50-75% of cases [3]. A high index of suspicion for dangerous or secondary causes is appropriate in the postpartum setting, where sinister features or “red flags” are similar to the non-pregnant patient (Table 1). 

**Table 1:** Headache "red flags".

Headache “Red Flags”
New headache, and not relieved by simple analgesia
Change in previously diagnosed headache
New focal neurology
Nausea and vomiting, worse when coughing or stooping over
Fever
Change in mental status with headache

Our patient presented with a sudden onset horizontal diplopia which was constant and worse on the left gaze. This was associated with headaches, which prompted further investigations to determine any secondary cause. Chambers DJ and Bhatia K [5] outline a useful list which we have modified (Table 2).

**Table 2:** Differentials of postpartum headache with cranial nerve palsy.

Differentials of postpartum headache with cranial nerve palsy
Space-occupying lesion
Intracranial tumour
Cerebral venous thrombosis
Subdural haematoma
Cavernous sinus thrombosis
Idiopathic intracranial hypertension
Cerebrovascular events such as stroke or posterior communicating artery aneurysm
Meningitis
Thyroid eye disease
Trauma
Post-dural puncture headache
Headache with co-existing disease, such as, multiple sclerosis, thyroid eye disease, myasthenia gravis, cranial nerve palsy (pre-existing)
Pre-eclampsia
Migraine

Most pathologies can be excluded by routine laboratory investigations and imaging, such as CT or MRI head. When these investigations return normal, and before considering more invasive investigations like lumbar punctures, it is important for the assessing team to recognise PDPH following epidural anaesthesia as a possible aetiology for a headache with an ocular nerve palsy in the postpartum period, particularly if the headache has the classic orthostatic features.

Epidural anaesthesia is a popular choice of pain relief during labour, with approximately 66% of deliveries requiring an epidural anaesthetic [6]. Spinal anaesthesia does not come without its risks. It poses risk for infection, post-dural puncture headaches and subdural haematomas which can be associated with cranial nerve palsies, and other neurological complications [7,8]. The estimated incidence of PDPH is <1% following neuroaxial anaesthesia in the obstetric setting [6,9].

Post-dural headache with cranial nerve palsy is even rarer. The incidence is variable, with some estimates being between 1 in 400 and 1 in 8000 of all spinal anaesthesia procedures [10]. Within obstetrics, a study documented one single case out of 27,000 births that developed a cranial nerve palsy [11]. As the rate is so low, it is seemingly unsurprising that an accurate true incidence has remained elusive, despite the first case being described over a century ago by Quincke [12].

A few articles highlight the presentation of cranial nerve palsies in the context of PDPH [5,10]. One case series reported that around 90% of cranial nerve palsies were unilateral, with the abducens and facial nerve being most commonly affected [5]. It is also understood that PDPH often occurs before diplopia [10]. The diplopia typically manifests one day to three weeks after the dural puncture procedure. The abducens nerve palsy is the most likely to completely resolve, with 89% of cases fully resolved between two weeks and eight months. Chronic cases lasting more than eight months are more likely to result in permanent diplopia [10].

The pathogenesis for PDPH and cranial nerve palsies is due to a cerebrospinal fluid (CSF) leak causing intracranial hypotension. The loss of CSF results in a reduced buoyance, venous engorgement and caudal displacement of the cerebrum, which puts traction on cerebral and cerebellar veins, meninges and cranial nerves. This explains the hallmark symptom of orthostatic headache, which worsens in an upright position and is alleviated when lying down. It can also be associated with other neurological problems such as impaired hearing, tinnitus, dizziness, meningism, diplopia, visual field defects and cranial nerve palsies [9], and rarely a subdural haematoma [13]. The preferential involvement of the abducens nerve in intracranial hypotension can be explained by understanding its anatomical course. In its course, there is an ascent just over the petrous bone which travels in the same direction as the caudal displacement of the brain noted in intracranial hypotension. This ultimately puts undue traction onto the nerve [5,10].

Imaging plays an important role in ruling out acute intracranial pathology, and detecting radiological signs of intracranial hyper- and hypotension. MRI has occasionally displayed markers of intracranial hypotension, such as small ventricles, diffuse post-contrast meningeal enhancement, downward displacement of the brainstem, subdural fluid collection and pituitary gland enlargement [14]. Diffuse pachymeningeal enhancement is one of the most common neuroimaging abnormalities related to intracranial hypotension [15].

Diplopia

Diplopia-related visits are common in an ophthalmology setting [16]. The majority of cases occur in elderly patients, over the age of 50 years [16]. When faced with a patient presenting with diplopia, taking a good history and performing a thorough examination can identify the cases that will require further investigations to rule out life-threatening pathologies (Table 3 and Table 4) for a quick summary. It is rare for postpartum patients with headaches and diplopia to present first to an ophthalmology department. In the rare instances when they do, the same steps should be followed in their assessment as for the general population, while ensuring to elicit a good obstetric history, with an emphasis on the use of epidural anaesthesia during labour.

**Table 3:** Key points in history and examination for diplopia.

Key points in history and examination for diplopia
1. Monocular vs Binocular
2. Constant vs Intermittent.
3.The orientation of the diplopia: horizontal, vertical or oblique – this can help identify the cranial nerve involved.
4. Prominence in different directions of gaze: constant vs worse in a certain position of gaze
5. Associated features: such as headache, pain on eye movement, ptosis, dysphagia, dyspnoea or any other focal neurology. In the elderly, it is particularly important to cover scalp tenderness, jaw or tongue claudication, temporal tenderness and constitutional symptoms. The presence of these should prompt investigations to rule out giant cell arteritis.
6. Any relevant past medical history such as hyper or hypothyroidism, myasthenia gravis, multiple sclerosis, cardiovascular disease or diabetes.

**Table 4:** Basic examination to assess diplopia. RAPD: Relative afferent pupillary defect.

Basic examination to assess diplopia
Assess visual acuity using a Snellen or LogMar chart
Assess pupils and look for an RAPD - presence of an RAPD suggests lesion anterior to the optic chiasm
Cranial nerve examination, particularly ocular motility - determine where the diplopia is the greatest, and if any pain exists. Multiple cranial nerve palsies is a sinister sign
Check for proptosis, ptosis, and lid fatigability

The first step in the assessment of a patient with diplopia is to distinguish if the condition is monocular or binocular. A binocular diplopia involves the perception of double vision only when both eyes are open and disappears when one eye is closed. It is a result of misalignment of the eyes and the visual axes, therefore, if acute, it must be considered as a neurological problem until proven otherwise. Binocular diplopia can be anatomically localised to supranuclear, internuclear, infranuclear, neuromuscular junction, extraocular muscle or orbital dysfunction [17]. Monocular diplopia, on the other hand, is still present with one eye closed (the unaffected eye) and suggests a pathology of the eye itself. In this instance, the patient should be referred to the Ophthalmology department for a complete eye examination. 

Once the diplopia is established as being binocular, it is important to elicit whether this is constant or intermittent. An intermittent diplopia implies that the diplopia is either situation-dependent or fatigable. An important consideration for diplopia that worsens with fatigue is myasthenia gravis. A decompensating long-standing squint can be intermittent as well, with the diplopia being worse when the patient is tired or following an illness. A constant diplopia should raise suspicion of a neurological issue.

A diplopia that does not change with the direction of gaze is considered comitant and would be suggestive of a decompensated congenital strabismus. Enquiring about the history of squint surgery or patching during childhood might help in establishing this diagnosis. Skew deviation should be considered if the diplopia is vertical and would suggest possible brainstem involvement. If the diplopia does change with the direction of gaze, it is described as incomitant. This is often due to extraocular muscle dysfunction. If the ocular movements are restricted in one of the classic patterns to suggest an oculomotor, trochlear or abducens nerve palsy (or a combination of those), then further investigations should be directed at finding a neurological cause. If no pattern can be identified, then orbital pathologies should be excluded. Examining for the presence of proptosis is important in these instances.

All patients presenting with diplopia require a full cranial nerves examination at a minimum, and ideally a full neurological examination. Any other focal neurology, the involvement of several cranial nerves or the presence of headaches or other neurological “red flags” could imply an intracranial pathology and would warrant further investigations to diagnose this. 

Management

The first-line treatment for PDPH is often caffeine which is effective in relieving the headache, unlike traditional analgesia. Caffeine blocks the adenosine receptors, causing arterial constriction and subsequently reducing intracranial blood flow and venous engorgement [18]. Bed rest and hydration are also common practices, as lying in a prone position aids with buoyancy and prevents further caudal traction by gravity.

An epidural blood patch has been shown to be highly effective for PDPH [6], with a documented success rate of up to 93% with the first attempt [10]. However, it has been less successful in treating cranial nerve palsies [5,10]. There have also been reported cases of cranial palsies occurring after administration of evidence-based practice (EBP) [5], but it is unclear if this is due to the EBP or perhaps the sequelae of intracranial hypotension following inadequate EBP administration. 

In this case, a blood patch was not offered as the neuropraxia already occurred and there was a high likelihood that the dural puncture would have already healed since the headache had settled by the time the patient was assessed at the maternity hospital. Therefore, the blood patch was deemed to carry more risk than benefit. MRI head and spine with gadolinium contrast was suggested as it would help determine if there were any obvious persisting CSF leaks and, if present, a blood patch would then possibly be indicated.

Treatment options for any ocular nerve palsies initially include eye patch and Fresnel prisms as a temporary measure. Surgical options for diplopia can be considered for permanent cases. It has been suggested that this should only be performed beyond 18 months, as there have been cases of diplopia taking up to 18 months to resolve [10].

## Conclusions

In this case, a postpartum patient presented with classic orthostatic headaches and an abducens cranial nerve palsy. An urgent CT scan without contrast did not reveal any acute intracranial pathology apart from an enlarged pituitary gland. Although this could be a sign of intracranial hypotension, an enlarged pituitary is common in the postpartum period and could therefore be unrelated. As the patient declined further imaging, a contrast CT or MRI was not performed to show any other features of intracranial hypotension. The diagnosis, in this case, was based on the clinical features alone in the context of the onset of symptoms shortly after an epidural anaesthetic.

This rare case report was taken from the unusual perspective of the ophthalmology department, where postpartum patients rarely present. We highlight to our fellow ophthalmology colleagues the importance of taking a good obstetric history and to enquire about the labour process with particular attention to the use of any epidural anaesthesia is of particular importance. We also stress the need to be familiar with postpartum conditions, and to be aware of certain postpartum conditions that can present with headaches and cranial nerve palsies such as cranial hypotension and subdural haematomas.

Equally, we underline the importance of distinguishing between monocular and binocular diplopia, and provide a basic framework in assessing diplopia. Finally, we emphasize the importance of good multi-disciplinary teamwork in rare complex cases in order to ensure patient safety and good patient care.
